# Supplementary material for: Determinants of utilization of cervical cancer screening among women in the age group of 30–49 years in Ambo Town, Central Ethiopia: A case-control study
Source: PLoS One. 2022 Jul 13;17(7):e0270821. doi: 10.1371/journal.pone.0270821 (PMC9278774; doi:10.1371/journal.pone.0270821)
Supplement: Attachment — Submitted filename: Response to Reviewers.docx [file pone.0270821.s002.docx]

**Author's response to Reviewers Comments**

Manuscript ID number: PONE-D-21-40695

**Title of Manuscript**: Determinants of Utilization of Cervical Cancer Screening among Women in the Age group of 30-49 Years in Ambo Town, Central Ethiopia, a case-control study

**Authors:**

1. Dereje Lema
2. Mecha Aboma: Corresponding Author: [abomamecha@gmail.com](mailto:abomamecha@gmail.com)
3. Teka Girma
4. Ababe Dechasa

**Generally, in manuscript we used track change to incorporate or address the reviewers’ comments.**

**A Point by Point Response to Reviewer Evaluation**

1. **Reviewer Question, #1:** Many grammatical errors are observed in the manuscript, the study is a case control study but in the conclusion section it is reported "Even though the facility of cervical cancer screening is available in almost all health facility levels but only a few of them utilized the services from existing data" this seems reporting prevalence of cervical cancer screening.

**Answers from authors:** The comment is accepted and corrected using track change and colored highlight throughout the manuscript, **Line 407-4025**

2. **Reviewer Question, #2:** The manuscript is reasonably sound, and the data support the conclusions presented. However, there are matters require revisions. The statistical analysis seems rigorous. The authors reported availability of data and promised that provide the data with reasonable request. The manuscript requires language revision.

**Answers from authors:** It is alright. As you indicate there are many errors. Using language expert we tried to correct using track change and colored highlight throughout the manuscript

3. **Reviewer Question, General comments:** This article tried to address important research question that is relevant and an interesting. The manuscript is generally well written and structured. However, in my opinion the paper has few shortcomings. For instance, research gap that authors would attempt to address in their project is not boldly stated.
   It needs to show gap and must attempt to fill in some piece of information missed in the scientific literature. Otherwise, it is not novel research which is not contributing to the overall goals of science.

**Answers from authors:** The comment is accepted and correction and revisions is made using track change and colored highlight throughout the manuscript.

4. **Reviewer Question, Title:** Determinants of Utilization of Cervical Cancer Screening among Women in the Age group of 30-49 Years in Ambo Town, Central Ethiopia: a case-control study. Why would you (authors) focus on this age group (30-49 years of age) as far as 15-49 years are also eligible?

**Answers from authors:** The government of Ethiopia launched screening of cervical cancer in 2014 and the VIA recommended for those women between the age of 30-49 years within every five years (6). Currently in Ethiopia cervical cancer screening services are free of charge in the public health facilities and women of 30-49 can uses services through self-referral, referral by health extension workers, by referral of other health care professionals (nurses, midwives, doctors ,and public health professionals) , and opportunistic screening.

5. **Reviewer Question, ABSTRACT: Background:** What stated in this section might not be research gap. It seems effort attempted by government to facilitate cervical cancer screening. What were known and not known since guidelines and protocols endorsed by government of Ethiopia?

**Answers from authors:** The comment is accepted and corrected, and indicated by track change and colored highlight, **Line 11-16**

6. **Reviewer Question, Methods:** “multivariate” should be edited and corrected as “Multivariable”

**Answers from authors:** The comment is accepted and corrected as “Multivariable”, **Line 20**

7. **Reviewer Question, Results:** it would be clearer if the total number of study participants involved were mentioned.

**Answers from authors:** The comment is accepted and corrected as **‘‘**a total of 195 study participants, sixty-five cases and one hundred thirty controls, were participated in this study making a response rate of 100%’’, **Line 22-23**

8. **Reviewer Question, Line 20.** Is grand multiparty appropriate instead of saying Para 5 and above?? It is optional.

**Answers from authors:** The comment is accepted

9. **Reviewer Question, Line 22:** high level of knowledge can be merged to the list of variables showed association with CCS. No need of separate explanation.

**Answers from authors:** The comment is accepted and corrected and indicated by using track change and colored highlight **Line 25-26**

10. **Reviewer Question, Conclusions:** I think it needs revision. It must be based on main findings.

**Answers from authors:** The comment is accepted and revision is made and indicated by using track change and colored highlight, **Line 407-4025**

11. **Reviewer Question, Line 27:** Why social media? Or would the authors say mass media? How many of women have access to social media? How feasible?

**Answers from authors:** The comment is accepted and corrected as ‘‘Mass media’’ **Line 31**

**INTRODUCTION:**

12. **Reviewer Question, Line 31:** It would be better if it changed to “introduction” than background. Because introduction consists of background and statement of the problem.

**Answers from authors:** The comment is accepted and corrected as ‘‘Introduction’’, Line 35

13. **Reviewer Question, Line 37-40:** these statements are not aligning with the prior statements and subsequent paragraph. The first paragraph state prevalence and death attributed to cervical Ca. The middle statements talking about methods of screening while the subsequent paragraph talking about Cervical Ca incidence by regions. Similarly, lines 103-108 are not appropriate place for this paragraph. Check it.

**Answers from authors:** The comment is accepted and revision is made and indicated by using track change colored highlight throughout the manuscript

14. **Reviewer Question, Line 111:** Dire Dawa is not a city administration. Rather, it is simply Dire Dawa Administration since it has the numbers of rural woredas and kebeles.

**Answers from authors:** The comment is accepted and corrected as ‘‘Dire Dawa Administration’’, **Line 147**

15. **Reviewer Question, METHODS and MATERIALS:**

Study setting- was missed.

This subtitle is important for what the authors stated from line 124-129. In addition, I personally want to incorporate other pertinent information such the numbers of health facilities, and trained health care providers who can provide CCS.

**Answers from authors:** The comment is accepted and corrected as: - ‘‘The town has one referral hospital, one general hospital, two health centers, nine health posts, and twenty one private clinics. Ambo General Hospital was the only hospital providing cervical cancer screening during this study was conducted. Ambo General Hospital had 287 health care workers. Those were 8 special doctors, 19 general practitioners, 69 nurses, and other health care providers (30).’’ **Line 168-172**. And study setting is added. **Line 62**

**Data collection tool and personnel**

16. **Reviewer Question, Line 154-158:** Here detail expiations about number of items used, response options, how composite scores made and relevant nature of tools, their validity are required. I suggest that merging and well synthesizing this section with operational definitions.
    **Answers from authors:** The comment is accepted and revision is made and indicated by using track change and colored highlight. Number of items also addressed under operational definition., **Line 198-204**
17. **Reviewer Question, Line 189:** there is phrase “overall knowledge”. What does it mean? Did the knowledge items have dimensions or domains? If that is the case define overall and Dimensions too.

**Answers from authors:** The comment is accepted and revision is made and indicated by using track change and colored highlighted, **Line 235-239**

18. **Reviewer Question**, Data management & Analysis: add versions of software used (EpiData and SPSS)

**Answers from authors:** The comment is accepted and corrected as ‘‘EPI-Data 3.1 version SPSS software version 25’’, **Line 2013-2014**

**RESULTS:**

19. **Reviewer Question, Line 215-216;** Either median with IQR or mean with SD is enough based distribution of the data

**Answers from authors:** The comment is accepted

20. **Reviewer Question,** Table 1: replace ‘’Socio-demographic characteristics of study participants (n=195)’’ with “variables”.

**Answers from authors:** The comment is accepted and indicated by using track change and colored highlighted, and characteristics replaced with ‘‘variables’’

21. **Reviewer Question, Line 226 and 229**: replace “have seen” with “saw” and “started” respectively.
    **Answers from authors:** The comment is accepted and indicated by using track change and colored highlighted, and “have seen” replaced with “saw” and “started” respectively.’’ **Line 273, 276**
22. **Reviewer Question, Line 234-235** remove “were

**Answers from authors:** The comment is accepted and corrected, and indicated by using track change and colored highlighted ‘’were’’ is removed, **Line 281, 282**

23. **Reviewer Question, Line 237:** replace “have” with “had”

**Answers from authors:** The comment is accepted corrected, and indicated by using track change and colored highlighted, and “have” replaced with “had” , **Line 284**

24. **Reviewer Question, Table 2:** variable “your husband has another wife”. It is not clear whether it is talking about polygamy or another wife or partner before she married him??
    **Answers from authors:** the comments accepted and corrected, and indicated by using track change and colored highlighted. It is talking about polygamy or currently having another wife
25. **Reviewer Question, Table 2:** “Number of partners”. It is not clear. Was it asking about number of partners in life time or current number of partners?
    **Answers from authors:** The comment is accepted and corrected, and indicated by using track change and colored highlighted. It is talking about number of partners in life time
26. **Reviewer Question, Knowledge related factors:** **Line 242-245:** The statements are too long, and vague/not clear. It was also mentioned under operational definitions that authors followed Bloom’s cut-off point to determine levels of knowledge. The same is true in lines 249-251.

**Answers from authors:** The comment is accepted and correction is made and indicated by using track change and colored highlighted, **Line 290-292, 296-298**

27. **Reviewer Question, Lines 255-265** need revision. Things already addressed in method section should be omitted. E.g., criteria set for candidacy to multivariable analysis.
    **Answers from authors:** The comment is accepted and correction is made and indicated by using track change and colored highlight, **Line 302-3013**
28. **Reviewer Question, Lines 270, 273, and 275:** The phrases “in the age group 30-49” are presented redundantly here and there unnecessarily. Since it was already mentioned in the title, no need of reporting them. Simply use “women who…….”

**Answers from authors:** The comment is accepted and correction is made and indicated by using track change and colored highlight, **Line 322, 324**

29. **Reviewer Question, Line 279**: “Towns”- delete “s”. Similarly, delete February to march” since it was mentioned in line 125.

**Answers from authors:** The comment is accepted and correction is made and indicated by using track change and colored highlight, **Line 328**

30. **Reviewer Question, Table 3**: under monthly income (>3000ETB) the corresponding confidence interval was 0.4-4. But to maintain consistency use at least one digit after decimal point. The same is true for high level of knowledge (2-17). It would be better it 2.0-17.0 if appropriate or subsequent number.

**Answers from authors:** The comment is accepted and correction is made and indicated by using track change and colored highlight

31. **Reviewer Question, DISCUSSION:** It would be appropriate if the first paragraph of discussion present summary of main findings. The main purpose of discussion is reporting findings one by one, interpreting, comparing with former findings and suggesting possible scientific justifications. In addition, explain newly emerged understandings from the current study.

**Answers from authors:** The comment is accepted and revision is made and indicated by using track change and colored highlight throughout the manuscript, **Line 334-338**

32. **Reviewer Question, This section needs strict revision, and arguments. Line 292**. It indicates misperception among younger women. Why that? Authors’ argument/ justifications should be scientific and strong enough to convince. Again, there are inconclusive findings? It is suggested that this must be exhaustively addressed and recommendations might be also required.

**Answers from authors:** The comment is accepted and revision is made and indicated by using track change and colored highlight as follow: - ‘‘Similarly, the explanation for this could be that of the bimodal distribution of cervical cancer, one in their 30s and the other in their 60s. These two age groups represent the ages at which cervical lesions become symptomatic.

Consequently, women see themselves as being at an increased risk of invasive cervical cancer as their age increases and seek health care and cervical cancer screening services. Additionally, in Ethiopia, the cervical cancer screening guideline promotes women aged 30–49 to be screened for cervical cancer, and women aged 40 and above might have better health-seeking behaviour and intention to be screened. Furthermore, this age group is more susceptible to giving birth at a productive age and has a chance of getting more gynaecological examinations, giving birth, and getting more health information about sexual and reproductive health, including cervical cancer screening services. The other explanation might also be that increasing risk with women’s age leads the women to have more contact with healthcare facilities.’’ **Line 346-459**

33. **Reviewer Question, Line 301-308:** it is wonderful ways of discussion.

**Answers from authors:** I thank you!

34. **Reviewer Question, Line 304:** Add Tanzania, after Dare Salam.

**Answers from authors:** The comment is accepted and Tanzania is added after Dare Salam **Line 369**

35. Reviewer Question, Lines 311-313: These kinds of stating justifications might be so superficial. It would better if authors specify reasons for difference than crudely reporting difference was due to “socio-demographic or sample size, time gaps”. Please, look them further in terms of design, inclusion criteria, age, awareness level etc.
    **Answers from authors:** The comments accepted and revision is made and indicated by using track change and colored highlight as follow: Differences in respondents' ages, levels of awareness, access to information such as mass media and other social media, family, peers, cultural beliefs, sociodemographic status, women's autonomy, economic conditions, physical and financial accessibility, disease patterns, and health service issues, as well as differences in the study design, study area, study period, study populations, and sample size, all contribute to the variation. **Line 377-383**
36. **Reviewer Question, CONCLUSIONS:** The first 3 lines (331-333) are enough. Recommendations are not appropriately made based main findings. For instance, statements in line 334 and 335(availability of the service was not studied in the current study. Three paragraphs starting from line 336-348 have no importance.

**Answers from authors:** The comment is accepted and correction is made and indicated by using track change and colored highlight, **Line 405-425**

**FIGURES:**

37. **Reviewer Question, Figure 1**: has no importance. I would suggest to omit it.

**Answers from authors:** The comment is accepted and the figure is removed

38. **Reviewer Question, Figure 3:** has miss classification in relation to what operationally defined.
    **Answers from authors:** The comment is accepted but the score/ or response for negative attitude was zero because all of the study participants has positive and neutral attitudes, (according to their response indicated)
39. **Reviewer Question, REFERENCES:**

• There is inconsistent way of reference writing. Some lacks volume, numbers, years etc.
• There are also old references published 10 years back. Unless special circumstances, it would better to use articles published since 2011 if guideline of journal allow.

**Answers from authors:** The comment is accepted, correction and revision is made and indicated by using track change and colored highlight

40. **Reviewer Question, OTHERS:** Minor editorial: punctuations, grammatical errors, inconsistent use terms

**Answers from authors:** The comment is accepted, correction and revision is made and indicated by using track change and colored highlight throughout the manuscript

41. **Reviewer Question, NOTE:** in conclusion, the findings of the manuscript are informative. But it requires moderate revision.

**Answers from authors:** The comment is accepted and revision is made and indicated by using track change and colored highlight throughout the manuscript.
