# Supplementary material for: Determinants of utilization of cervical cancer screening among women in the age group of 30–49 years in Ambo Town, Central Ethiopia: A case-control study
Source: PLoS One. 2022 Jul 13;17(7):e0270821. doi: 10.1371/journal.pone.0270821 (PMC9278774; doi:10.1371/journal.pone.0270821)
Supplement: Attachment — Submitted filename: Response to Reviewers.docx [file pone.0270821.s003.docx]

**Author's response to Reviewers and Academic Editors Comments**

Manuscript ID number: PONE-D-21-40695

**Title of Manuscript**: Determinants of Utilization of Cervical Cancer Screening among Women in the Age group of 30-49 Years in Ambo Town, Central Ethiopia, a case-control study

**Authors:**

1. Dereje Lema
2. Mecha Aboma: Corresponding Author: [abomamecha@gmail.com](mailto:abomamecha@gmail.com)
3. Teka Girma
4. Ababe Dechasa

**Generally, in manuscript we used track change to incorporate or address the reviewers’ comments.**

**A Point by Point Response to academic editor and reviewer(s) Evaluation**

1. **Academic Editors Question, #1:** Please review your reference list to ensure that it is complete and correct. If you have cited papers that have been retracted, please include the rationale for doing so in the manuscript text, or remove these references and replace them with relevant current references. Any changes to the reference list should be mentioned in the rebuttal letter that accompanies your revised manuscript. If you need to cite a retracted article, indicate the article’s retracted status in the References list and also include a citation and full reference for the retraction notice.

**Answers from authors:** The comment is accepted and corrected using track change and colored highlights throughout the manuscript. However, we didn’t use retracted and unpublished papers in the references, all of the references we used in this manuscript are available on Search Google or/and Google Scholars.
